# Role of Methanesulfonic Acid in Sulfuric Acid–Amine and Ammonia New Particle Formation

**DOI:** 10.1021/acsearthspacechem.3c00017

**Published:** 2023-03-07

**Authors:** Jack S. Johnson, Coty N. Jen

**Affiliations:** † Department of Chemical Engineering, Carnegie Mellon University, Pittsburgh, Pennsylvania 15213, United States; ‡ Center for Atmospheric Particle Studies, Carnegie Mellon University, Pittsburgh, Pennsylvania 15213, United States

**Keywords:** nucleation, new particle formation, sulfuric acid, methanesulfonic acid, amines, atmospheric aerosols, marine atmosphere

## Abstract

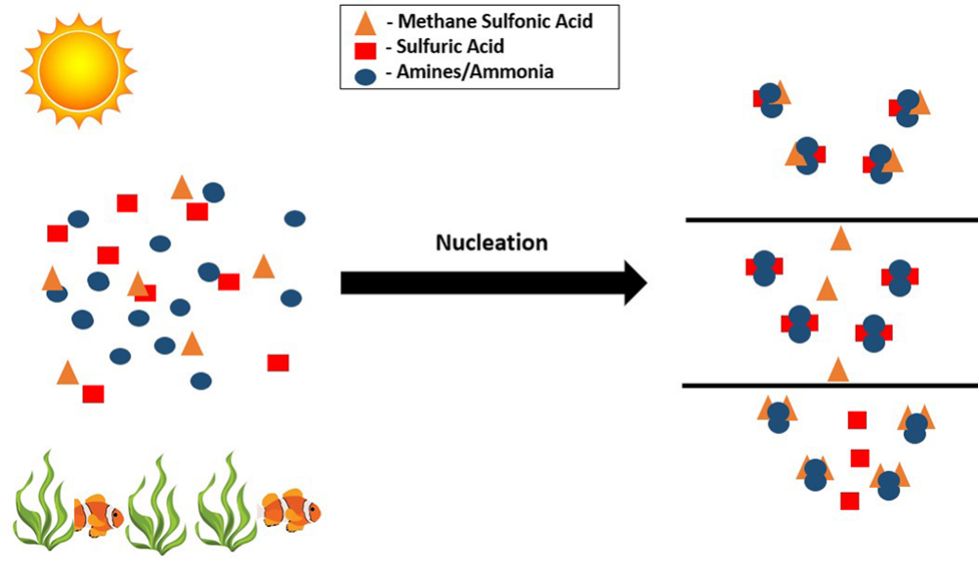

Aerosol nucleation accounts for over half of all seed particles for cloud droplet formation. In the atmosphere, sulfuric acid (SA) nucleates with ammonia, amines, oxidized organics, and many more compounds to form particles. Studies have also shown that methanesulfonic acid (MSA) nucleates independently with amines and ammonia. MSA and SA are produced simultaneously via dimethyl sulfide oxidation in the marine atmosphere. However, limited knowledge exists on how MSA and SA nucleate together in the presence of various atmospherically relevant base compounds, which is critical to predicting marine nucleation rates accurately. This work provides experimental evidence that SA and MSA react to form particles with amines and that the SA-MSA-base nucleation has different reaction pathways than SA-base nucleation. Specifically, the formation of the SA-MSA heterodimer creates more energetically favorable pathways for SA-MSA-methylamine nucleation and an enhancement of nucleation rates. However, SA-trimethylamine nucleation is suppressed by MSA, likely due to the steric hindrance of the MSA and trimethylamine. These results display the importance of including nucleation reactions between SA, MSA, and various amines to predict particle nucleation rates in the marine atmosphere.

## Introduction

Particle nucleation in the atmosphere may impact cloud formation and the Earth’s radiation balance.^1,2^ Atmospheric nucleation occurs when gas-phase compounds react to form a stable cluster. Sulfuric acid (SA, H_2_SO_4_), an oxidation product of sulfur dioxide (SO_2_) and dimethyl sulfide (DMS, C_2_H_6_S), has been shown to nucleate in the atmosphere, and its concentration in the atmosphere typically correlates with particle nucleation rates.^3−9^ While sulfuric acid is an important molecule for atmospheric nucleation, sulfuric acid concentration alone cannot explain observed particle nucleation rates.^3,10^ Various compounds can react with sulfuric acid to form particles, including ammonia^11−13^ and amines.^14−16^ These basic compounds are found in the atmosphere and are emitted through biomass burning, animal husbandry, and chemical and industrial plants.^17^ For sulfuric acid–amine systems, extensive work has been conducted to determine the acid–base reaction steps for forming these particles.^3,16,18−20^ In addition, sulfuric acid–base nucleation has been observed in ambient air, and its rates are quantified in the atmosphere worldwide.^5,21−24^ As sulfuric acid nucleation is an integral part of weather and climate; recent studies have incorporated sulfuric acid–ammonia and amine nucleation into global climate models to improve predictions of atmospheric aerosol number concentrations.^25−27^

In addition to sulfuric acid, recent studies have found that methanesulfonic acid (MSA, CH_4_O_3_S) also contributes to particle nucleation and growth in the atmosphere.^28,29^ MSA is primarily found in coastal and oceanic regions^29^ as it is an oxidation product of DMS, a marine emission.^30,31^ Amines and ammonia are also emitted in a marine environment, mainly from phytoplankton.^32−34^ Previous field measurements indicate that MSA exists at around 10–100% of SA concentration,^28,35^ with laboratory measurements showing MSA can nucleate with amines like dimethylamine (DMA, (CH_3_)_2_NH).^36^ Despite MSA’s importance in atmospheric nucleation and its prevalence in the marine environment, no global climate models currently account for MSA nucleation.

While SA, MSA, amines, and ammonia coexist in a marine atmosphere, limited information exists on how these compounds nucleate together to form particles. Chen et al.^37^ have demonstrated that MSA-amine reactions can form particles at parts per billion level concentrations of reactants, higher than measured in the atmosphere.^37^ In addition, Elm’s computational chemistry results previously showed that the inclusion of MSA to the SA-base system creates a strong interaction between MSA, SA, and ammonia/amines that could potentially enhance the nucleation rates compared to the SA-base systems.^38,39^ However, this study was limited to clusters containing up to two acid molecules, any number of base molecules, and no water. While these cluster binding energies suggest that MSA could influence sulfuric acid nucleation rates, experimental observations are required to uncover the dominant stepwise reactions between MSA, SA, and amines/ammonia.

The experimental study presented here examines the nucleation reactions in the SA-MSA-amine/ammonia systems. Mass spectrometer measurements of freshly nucleated clusters show that MSA is involved in the first steps of nucleation. Particle concentration measurements also show that MSA could enhance or suppress sulfuric acid–base particle formation rates depending on the base compound. Results demonstrate that the role of MSA in MSA-SA-base in new particle formation, i.e., nucleation and growth, is dependent on the ratio of SA and MSA concentrations and the interaction of MSA with the various basic compounds. Including MSA when modeling atmospheric new particle formation, especially in marine environments, is needed to accurately predict particle concentrations in the atmosphere.

## Methods

Nucleation experiments were conducted using a clean and repeatable glass flow reactor as described in Fomete et al.,^40^ with pertinent details repeated here. The flow rate and temperature of the reactor are held constant at 4 LPM and 298–300 K (based on small fluctuations in room temperature), respectively, while relative humidity (RH) is 20%. There are three main injection flows into the flow reactor: nitrogen entrained with sulfuric acid, dry nitrogen, and humidified nitrogen. Sulfuric acid vapor is generated by passing nitrogen over a liquid sulfuric acid reservoir and injected at the top of the reactor. Sulfuric acid concentration is controlled by specifying the flow rate through the reservoir with concentrations ranging from 10^7^ to 10^9^ cm^–3^. Humidified nitrogen and dry nitrogen streams are also injected into the top of the reactor to control the RH in the flow reactor and provide a dilution stream for sulfuric acid. The reactor has been continuously purged for ∼2 years with gaseous sulfuric acid, nitrogen, and water to remove potential contaminant compounds and ensure repeatable reaction conditions.^16,40,41^ Baseline measurements of the sulfuric acid dimer (i.e., a cluster containing two sulfuric acid molecules and any water molecules that evaporate upon measurement) and particle concentrations as a function of sulfuric acid concentration are taken daily to ensure consistent measurements across all experiments.^40^ MSA was injected into the flow reactor at 80–120 sccm, and its concentration was varied from 10^7^ to 10^10^ cm^–3^ by adjusting the flow rate of N_2_ over the liquid MSA reservoir. Gaseous ammonia (NH_3_, Wards Science 40 wt % in H_2_O), methylamine (MA, CH_3_NH_2_, Sigma-Aldrich 40 wt % in H_2_O), dimethylamine (DMA, (CH3)_2_NH, Acros Organics 40 wt % in H_2_O), and trimethylamine (TMA, C_3_H_9_N, Sigma-Aldrich 45 wt % in H_2_O) were generated and injected into the flow reactor using custom-built permeation tubes and a double dilution system.^40,42^ No unexpected or unusually high safety hazards were encountered.

For the nucleation experiments, MSA was injected into the flow reactor, allowing SA and MSA to mix for ∼8 s. Amines/ammonia were injected into the flow reactor and allowed to nucleate with MSA and SA for ∼2 s prior to measurement. This nucleation time is based on the centerline velocity from the flow parameters and distance to the measurement point.

Concentrations of MSA, SA, and the base compounds, as well as freshly formed molecular clusters, are measured using an atmospheric pressure chemical ionization quadrupole mass spectrometer known as the Minnesota Cluster CIMS (MCC).^16,43−45^ Acetate and nitrate were used as reagent ions to ionize acidic molecular clusters. Nitrate was used for dimer cluster observations and most particle observations. Acetate ionization was used to measure SA and MSA concentrations during the particle observations for MA and TMA when varying [MSA]. The reaction rate constant was 2 × 10^–9^ cm^3^ s^−1^ for nitrate and 4.6 × 10^–9^ cm^3^ s^−1^ for acetate.^46−48^ Additionally, it is assumed that MSA is ionized by nitrate and acetate at the same rate as SA, as no previous measurements have been conducted on its ionization rate constant. Hydronium ions (and its larger clusters) were used to ionize the basic gases. Ion signals are converted to concentrations using the method described in Fomete et al.,^48^ with a chemical ionization time of 0.02 s. Mass-dependent transmission efficiency values for the MCC were used to account for differences in the detection due to ion mass.^45^ The systematic uncertainty of the MCC has been estimated to be a factor of two.^14^ However, this uncertainty would affect all measurements equally and thus would have little impact on the overall trends in the data.

Particle concentrations were measured with a 1 nm versatile water condensation particle counter (vwCPC, TSI 3789).^49^ The conditioner on the vwCPC was set to 1 °C, and the initiator was set to 99 °C. The nucleation time of 2 s was chosen to ensure that freshly formed particles did not grow beyond ∼1 nm in diameter during the short nucleation time. Longer nucleation times would result in higher coagulation losses, which would obscure the reaction formation pathways. In comparison, shorter nucleation times would mean particles are smaller than 1 nm and would not get measured by the vwCPC. Figure S1 shows the difference in particle counts of the vwCPC at a 1 nm setting vs the 2 nm setting (conditioner set to 2 °C and the initiator set to 90 °C) while injecting 7 pptv of TMA and [MSA] = 4 × 10^9^ cm^–3^ into the sulfuric acid flow reactor. Particle concentrations decreased by over 97% when changing the vwCPC temperatures from the 1 nm to the 2 nm setting. The decrease in particle concentration indicates that almost all of the particles formed are 1–2 nm.

## Results and Discussion

### Dimer Cluster Observations

**Figure 1:**
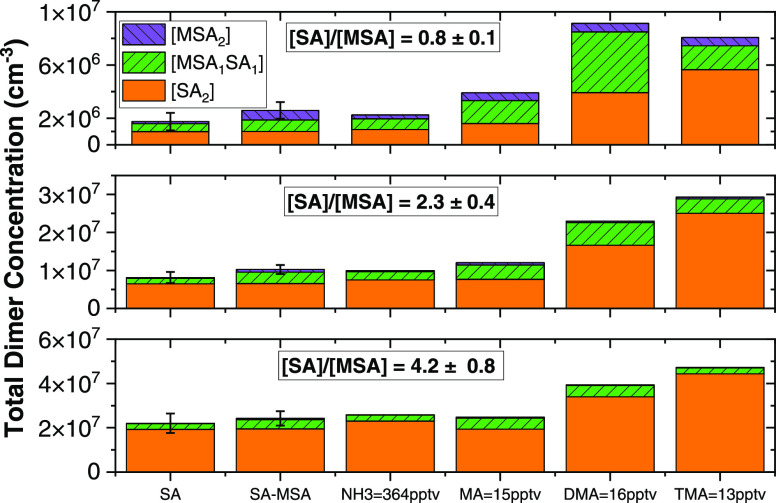
Comparison of the total concentrations of sulfuric acid (SA) and methanesulfonic acid (MSA) dimers with various base compounds. Each panel represents an increasing ratio of [SA]/[MSA], with [MSA] = 2 × 10^8^. Solid orange represents [SA·SA], green with forward slash lines is [MSA·SA], and purple with back slash lines is [MSA·MSA]. SA bar shows the background concentrations of dimers when no MSA or base is injected into the flow reactor. SA-MSA bar is the system without a base. The remaining bars correspond to the SA+MSA+ base systems. The uncertainty bars represent the variations in concentrations of dimers across each set of experiments.

Dimer concentrations measured by the MCC using nitrate ionization include the SA dimer ([SA·SA]), MSA dimer ([MSA·MSA]), and heterodimer ([SA·MSA]). Dimer clusters likely had water and base molecules attached that evaporated upon measurement.^50^ Throughout the discussion of monomers and dimers, the convention used is a monomer or dimer refers to the number of acid molecules in the cluster rather than the total number of molecules. An increase in acid dimer concentrations is a useful indicator of particle formation, and likely, the acid dimers contained a base molecule that evaporated when the cluster was ionized. Figure 1 shows measured dimer concentrations in the sulfuric acid flow reactor upon adding MSA, DMA, TMA, MA, and NH_3_ at RH = 20%. Each panel illustrates how dimer concentrations change when [SA] is roughly equal to and greater than [MSA] = 2 × 10^8^ cm^–3^ and the base concentration is constant. Uncertainty bars for SA and SA-MSA with no base addition represent the standard deviation in the background [SA·SA], [MSA·SA], and [MSA·MSA]. This standard deviation was calculated over the four experimental days for each base compound. [MSA·SA] during the SA background measurements is 11, 18, and 34% of the total dimer concentration with decreasing [SA]. While the fraction of [MSA·SA] does increase at low [SA]/[MSA], the [MSA·SA] = 6 × 10^5^ cm^–3^ is low when compared to the SA-MSA injections. The [MSA·SA] during the SA background measurements is likely due to trace amounts of contamination of MSA (∼8 × 10^7^ cm^–3^) carried over from previous experiments. The addition of MSA to a pure SA (and water) system does not significantly impact [SA·SA], with the [SA·SA] concentration increasing by approximately 2 × 10^5^ cm^–3^ (1.3%) for high [SA]/[MSA], 6 × 10^4^ cm^–3^ (1.1%) for medium [SA]/[MSA], and 1 × 10^3^ cm^–3^ (0.2%) for low [SA]/[MSA]. This negligible increase in [SA·SA] concentrations indicates that there are little to no molecular interactions occurring between acid molecules when no base is present in the reactor. The SA·SA dimer without a base is likely forming via ion-induced clustering (IIC) as the binding free energy of the uncharged cluster is weak at −5.5 kcal/mol.^45^ IIC occurs within the inlet of the MCC and is where ions that have been electrically charged by the reagent ion (e.g., nitrate) continue to react and form clusters with other neutral molecules within the flow reactor.^40^ Similarly, MSA·SA and MSA·MSA may also form via IIC in the SA and MSA injection conditions. IIC likely influences all of the dimers as SA·SA, MSA·SA, and MSA·MSA have similar computed binding free energies of −5.5, −5.1, and −5.4 kcal/mol, respectively. Note that all referenced binding free energies are provided by Elm^38,39^ and summarized in Table S1. [MSA·MSA] remains low and unchanged for all of the base molecules, implying that either the MSA·MSA·base or MSA·base clusters are unstable at 298–300 K. In addition, Elm^39^ has shown that the computed MSA·base free energies range from −3.4 to −8.7 kcal/mol.^39^ In contrast, SA·base free energies are generally stronger, ranging from −5.6 to −12.6 kcal/mol.^39^ Interestingly, the [MSA·MSA] and [MSA·SA] are lower than [SA·SA] when [SA]/[MSA] ∼ 1, even though the free energies of the uncharged clusters are similar. Note that when [SA] is approximately equal to [MSA], a cluster has an equal probability of colliding with either a SA or MSA molecule. It may be possible that MSA does not ion-induce a cluster with MSA or SA as effectively as negatively charged SA with SA. However, MSA could still be participating in IIC, which would explain the higher ratios of the MSA·MSA and MSA·SA clusters when [SA]/[MSA] ∼ 1. Regardless, the trends show that MSA appears to have little impact on the formation of the dimers when no base is present.

Figure 1 also shows the [SA·SA], [MSA·MSA], and [MSA·SA] when injecting amines and ammonia. Likely, many of the dimers formed in Figure 1 contained a base molecule before ionization, as pure acid dimers are unstable at 298–300 K. Dimer concentrations generally increase compared to the SA and SA-MSA measurements when a base compound is present, indicating the base compounds are reacting to form acid dimers. In the case of NH_3_ addition, the total dimer concentration increased by 4% at high [SA]/[MSA] but decreased by 13% at low [SA]/[MSA]. The slight decrease in dimer concentration is within the uncertainty at low [SA]/[MSA] where there is greater fluctuation in [SA] due to small changes in flow rate for sulfuric acid. Additionally, due to the relatively weak binding interactions between SA and NH_3_, there is likely minimal dimer formation at lower [SA]/[MSA], which is evident by the low [SA·SA] of 1 × 10^6^ cm^–3^. The dimer diversity is especially noticeable when the [SA]/[MSA] ∼ 1 in the top panel. MSA·SA and MSA·MSA become a more significant fraction of the total dimer concentration, especially with the addition of either DMA or MA. The increase in MSA-containing dimers indicates that the amine is reacting with MSA and SA to enhance the formation of MSA·SA and MSA·MSA. As previously mentioned, MSA·SA and MSA·MSA quickly evaporate at 298–300 K. Thus, the amine molecule likely attaches to SA and MSA prior to reacting with another SA or MSA to form a stable dimer.

The [SA·SA] displayed in Figure 1 is slightly higher ([SA·SA] = 1.9 × 10^7^ cm^–3^ for SA, and [SA·SA] = 2.3 × 10^7^ cm^–3^ for SA-MSA-NH_3_) between SA and SA-MSA-NH_3_ systems, showing that SA-MSA-NH_3_ nucleation forms weakly bonded clusters, similar to SA-NH_3_ nucleation.^14,51,52^ The [MSA·SA] and [MSA·MSA] also do not vary between the SA-MSA and SA-MSA-ammonia systems, indicating that ammonia does not help form stable MSA clusters. Also, increasing the [SA]/[MSA] ratio does not alter the dimer concentrations compared to the no ammonia addition case. This result signifies ammonia does not preferentially prefer to react with SA or MSA and agrees with computed binding free energies, where SA·SA·NH_3_ is −19.4 kcal/mol and MSA·SA·NH_3_ is −18.3 kcal/mol.^38^ The binding free energies for SA·SA·NH_3_·NH_3_ and MSA·SA·NH_3_·NH_3_ are −27.0 and −23.6 kcal/mol, respectively.^38^ The more strongly bonded SA·SA·NH_3_·NH_3_ cluster indicates that ammonia shows some preference to form SA dimer and could explain the slightly higher [SA·SA] compared to SA and SA-MSA systems.

For MA = 15 pptv in Figure 1 at [SA]/[MSA] ∼ 1, the [MSA·SA] and [SA·SA] are approximately equal. The similar [MSA·SA] and [SA·SA] in this regime then suggest the stability of the MSA·SA·MA and SA·SA·MA clusters are roughly equal, or the stability of the monomers (SA·MA and MSA·MA) are similar. The binding free energies of SA·SA·MA is −24.4 and −24.2 kcal/mol for MSA·SA·MA.^38,39^ In contrast, the free energies of the monomers are not similar as SA·MA is more strongly bonded at −7.2 kcal/mol when compared to MSA·MA at −3.9 kcal/mol.^38,39^ These free energies suggest that [MSA·SA] and [SA·SA] are equal when [SA] ∼ [MSA] because MA first reacts with SA before adding either SA or MSA. In addition, MSA·SA·MA·MA and SA·SA·MA·MA have similar binding free energies of −33.8 and −36.6 kcal/mol respectively, which are both stable. As the [SA]/[MSA] ratio increases, [SA·SA] also increases. This trend is expected since a cluster is more likely to collide with SA in this high SA regime, and MA has previously been shown to help form relatively stable sulfuric acid clusters.^14,16,53^

For DMA = 16 pptv in Figure 1, the [SA·SA] and [MSA·SA] increase by over 50% compared to the no DMA system. The higher [SA·SA] is not surprising, given DMA’ss well-established high stability effects on sulfuric acid clusters.^16^ For [SA]/[MSA] ∼ 1, the [SA·SA] = 3.0 × 10^6^ cm^–3^ and [MSA·SA] = 4.6 × 10^6^ cm^–3^, which suggests similar stabilities of MSA·SA·DMA and SA·SA·DMA clusters. The similar stabilities of these two clusters are also confirmed by Elm’s^34^ computed binding free energies, where SA·SA·DMA and MSA·SA·DMA are roughly equal at −29.4 and −28.2 kcal/mol, respectively.^38^ Furthermore, the fraction of [MSA·SA] out of the total dimer concentration decreases with increasing [SA]/[MSA], even though the total dimer concentration is increasing. The [SA·SA] also increases by an order of magnitude with higher [SA]/[MSA]. These combined observations suggest that the SA·DMA cluster is more stable than MSA·DMA, which is confirmed by previously computed binding free energies of −11.5 and −7.1 kcal/mol.^38,39^ Thus, DMA will preferentially form dimers faster with SA than MSA.

**Figure 2:**
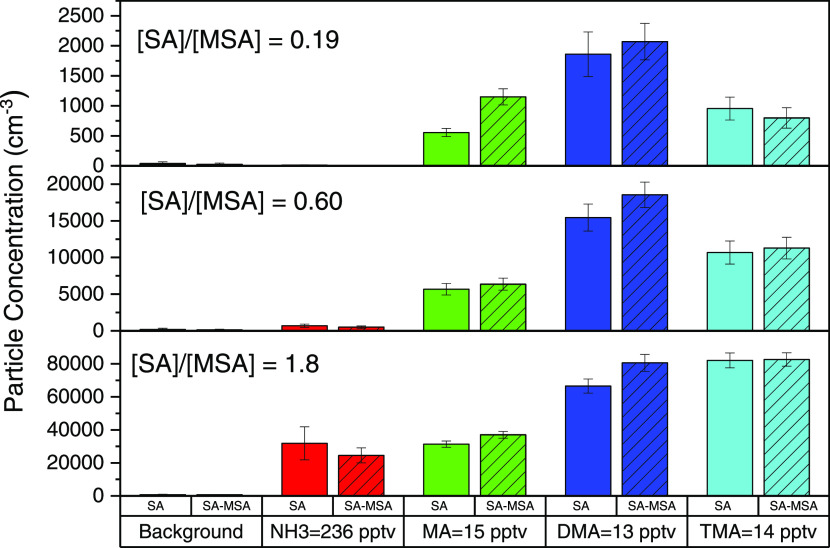
Comparison of total particle counts after 2 s nucleation time with various bases. Solid colors indicate SA-base nucleation, and solid colors with slashed lines indicate SA-MSA-base nucleation, where MSA = 2.5 × 10^8^ cm^–3^. Black bars show background particle counts, red is ammonia at 236 pptv, green is DMA at 13 pptv, dark blue is MA at 15 pptv, and light blue is TMA at 14 pptv. Error bars provide the standard deviation in particle concentrations across each set of experiments.

At TMA = 13 pptv in Figure 1, the total dimer concentration is similar to the DMA-MSA-SA system at each ratio of [SA]/[MSA]. The [MSA·SA] is lower with TMA than DMA across the studied [SA]/[MSA] regimes. TMA likely reacts with MSA-containing clusters to a lesser extent compared to DMA. This result agrees with binding free energies where MSA·SA·DMA is −28.2 kcal/mol and MSA·SA·TMA is −24.9 kcal/mol. SA·TMA is also more strongly bonded (−12.6 kcal/mol) than MSA·TMA (−8.7 kcal/mol).^38^ Additionally, there is a significant decrease in the binding free energy of MSA·SA·TMA·TMA (−31.9 kcal/mol) when compared to SA·SA·TMA·TMA (−41.5 kcal/mol). This decrease in the free energy for MSA·SA·TMA·TMA indicates that at larger cluster sizes, TMA is much more likely to react with SA, which matches the high fraction of [SA·SA] for TMA in Figure 2. Therefore, the main pathway for dimer formation for the TMA-MSA-SA system is TMA reacting with SA.

Comparison of the observed dimer concentrations of the TMA-SA-MSA and MA-SA-MSA systems leads to disagreement with computed free energies. Specifically, MSA·SA·TMA has similar binding free energies as MSA·SA·MA (−24.9 and −24.2 kcal/mol, respectively). Furthermore, TMA has stronger binding free energies with MSA than MA (−8.7 and −3.9 kcal/mol, respectively). Despite the overall stronger energies of TMA with MSA and SA, the [MSA·SA] in the TMA system is roughly half of the [MSA·SA] in the MA system when [SA]/[MSA] ∼ 1. The unexpectedly lower [MSA·SA] with TMA suggests that steric hindrance is preventing the formation of MSA·TMA or MSA·SA·TMA, which are not captured in the computed binding free energies. In addition, the total dimer concentration of TMA is the highest of all bases at high [SA]/[MSA], while DMA has the highest total dimer concentration when [SA]/[MSA] ∼ 1. TMA’s more significant reduction in total dimer concentration at [SA]/[MSA] ∼ 1 could also suggest possible steric hindrance effects between TMA and MSA. This possibility is further explored below in the particle measurements.

**Scheme 1:**
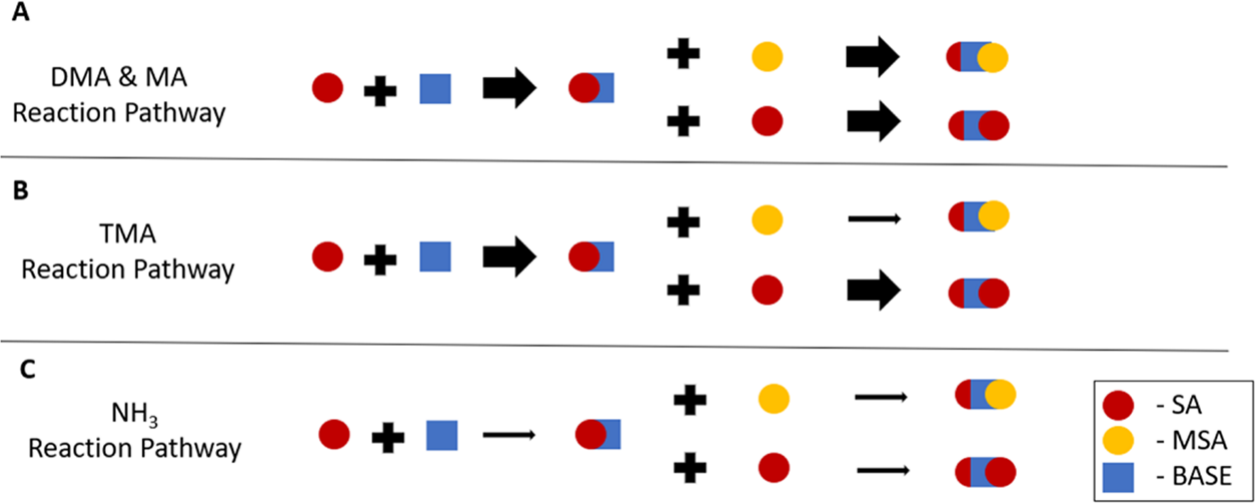
Summary of Reaction Pathways Each symbol represents a molecule of sulfuric acid (red circle), methanesulfonic acid (yellow circle), and base (blue square). The arrow’s width indicates the likelihood of that pathway for the reaction to occur when [SA] ∼ [MSA]. Panel A shows the formation pathways for DMA and MA. Panel B for shows formation pathways for TMA, and Panel C shows the pathways for NH_3_.

Scheme 1 summarizes the reaction pathways for the SA-MSA-base system. The likely formation pathway for these amines/ammonia starts with the reaction of SA and base. For DMA and MA, the monomer adds either a SA molecule or an MSA molecule at similar rates. From Scheme 1B, the first step is still the addition of a TMA molecule to SA; however, for TMA, the more energetically favorable next step is the addition of SA instead of MSA. This reaction pathway is consistent with the cluster observations from Figure 1 that show a significant increase in the [SA·SA] when TMA is present in the flow reactor. Scheme 1C shows how ammonia has relatively weak interactions with both acid molecules and likely only slightly prefers reacting with SA.

The combined observations demonstrate that MA and DMA can form stable clusters with MSA. Ammonia is likely too weak of a stabilizing base to cluster with MSA. TMA showed an appreciable increase in [MSA·SA] compared to no TMA, but [SA·SA] still dominates the total dimer concentration. Thus, TMA likely reacts primarily with SA. This result is possibly due to TMA’s greater steric hindrance that does not allow TMA to react as readily with MSA beyond the monomer.

Clusters larger than the dimer were observed in the MSA-SA-base systems. Small amounts of SA timer and tetramer were observed for the amines/ammonia. However, there were likely other trimers that included amines and MSA that were not being measured due to cluster fragmentation inside the MCC or during the atmospheric ionization with nitrate. Future work should explore cluster ionization to better understand how to measure larger molecular clusters.

### Particle Observations

Since clusters are larger than the dimer fragment in the MCC, particle measurements were taken with the vwCPC for the SA-MSA-base systems to better understand nucleated particles’ formation rates. Figure 2 shows the particle concentrations from SA-MSA, SA-base, and SA-MSA-base nucleating systems. For the particle observations, the ratios of [SA]/[MSA] were generally lower than for the dimer cluster measurements (Figure 1) to avoid the saturation limit of the vwCPC (1 × 10^5^ cm^–3^). For all [SA]/[MSA], particle concentrations increase or decrease by approximately 20–30% when injecting MSA, with the largest difference being 202–253 cm^–3^ when injecting MSA. These changes in particle concentrations are not significantly different when comparing SA and SA-MSA systems. The insignificant change in particle concentrations when injecting MSA agrees with the dimer observations of Figure 1, where SA-MSA does not form an appreciable number of dimers and thus does not form particles. In addition, at high [SA]/[MSA], the observed particle concentrations between the SA-MSA-base systems are similar. However, particle concentrations exhibit significant differences at a lower ratio of [SA]/[MSA], specifically for MA and TMA. At low [SA]/[MSA], clusters are more likely to collide with MSA instead of SA, and thus this regime focuses on the influences of MSA on new particle formation. For MA, there is an increase in particle concentrations in the SA-MSA system when compared to SA, while for TMA, there is a decrease in particle concentrations with SA-MSA. MA and TMA will be explored further below to determine MSA’s overall impact on their reaction pathways.

**Figure 3:**
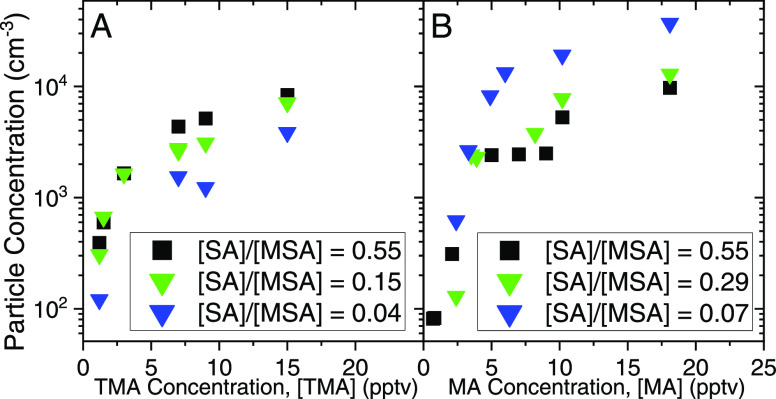
(A) SA-MSA-TMA nucleation and (B) SA-MSA-MA nucleation, where [SA] = 5 × 10^7^ cm^–3^, with a nucleation time of 2 s. Black squares are [MSA] ∼ 1 × 10^8^ cm^–3^, green triangles are [MSA] ∼ 2 × 10^8^ cm^–3^, and blue triangles are [MSA] ∼ 1 × 10^9^ cm^–3^.

Following experiments where [SA] and [MSA] are changed, [TMA] was also varied to quantify the suppression of new particle formation at high [MSA]. Figure 3A shows the particle concentration results from the SA-MSA-TMA system. MSA and TMA concentrations were varied here, and [SA] = 5 × 10^7^ cm^–3^. Each curve represents a different ratio of [SA]/[MSA] from 0.55 to 0.04 for TMA. In Figure 3A, at high [TMA] = 15 pptv, the particle concentrations drop by approximately 50% when [MSA] concentration increases by an order of magnitude. Furthermore, Figure S2 shows that this decrease in particle counts was due to MSA suppressing SA-TMA nucleation and not coagulation. The observed decrease in particle concentration is likely due to the steric hindrance in the SA-MSA-TMA system, where MSA binds up available TMA (as MSA·TMA), preventing further reaction with MSA or SA. This result also agrees with the dimer cluster observations (Figure 1) that showed significantly less [MSA·SA] for TMA than other strong stabilizing amines like DMA. While computational results show strong binding free energies for SA-MSA-TMA clusters, steric hindrance that lowers the collision accommodation coefficient (i.e., sticking coefficient) is not captured by these *ab initio* calculations. MSA suppressing SA-TMA nucleation is a significant result, as there has been limited information in previous literature about compounds capable of suppressing SA nucleation.

Figure 3B shows the particle concentration results for the SA-MSA-MA system. MSA and MA concentrations were varied with [SA] = 5 × 10^7^ cm^–3^. Each curve represents a different ratio of [SA]/[MSA] from 0.55 to 0.07 for MA. Figure 3B shows the opposite phenomena to Figure 3A, where increasing [MSA] by an order of magnitude leads to an increase in the particle concentrations up to a factor of three. The increase in particle concentrations signifies that MA can nucleate with MSA and SA. MA likely nucleates with both acids because MA is a significantly smaller molecule than TMA and less likely to be sterically hindered when colliding and reacting with MSA. MSA reacting with MA to form particles is also in agreement with computational chemistry results that showed equal stabilities of the SA·SA·MA cluster and the MSA·SA·MA cluster.

Particle concentrations for the SA-MSA-MA system are still lower than SA-MSA-TMA until [SA]/[MSA] < 0.55. This difference between base systems is due to the first step in monomer cluster formation, which is likely the new particle formation rate-limiting step. Though MA can add to MSA and SA, these clusters (SA·MA and MSA·MA) are still more weakly bonded than those with TMA (specifically SA·TMA). Thus, while MA enhances particle formation rates for the SA-MSA system, TMA remains a potent nucleating compound with SA and still plays an important role in atmospheric marine nucleation.

## Conclusions

Methanesulfonic acid (MSA) impacts the initial steps of cluster formation in the SA-MSA-base system. Specifically, adding MSA to the SA-base system allows more pathways for dimer cluster formation, as indicated by the formation of the MSA dimer and the SA-MSA heterodimer. The fraction of [SA·SA], [MSA·MSA], and [SA·MSA] formed out of the total dimer concentration also indicates which base compounds are reacting more readily with MSA than others. Methylamine and dimethylamine react with SA and MSA to form relatively equal ratios of [MSA·SA] and [SA·SA], indicating that MSA is readily reacting with MA and DMA. However, [MSA·SA] in the case of TMA is significantly lower than the [SA·SA], indicating that TMA is preferentially nucleating with SA. There are only minor increases to dimer concentrations for ammonia, indicating that any interactions between ammonia and MSA are likely negligible compared to SA and ammonia.

Particle measurements showed that while MSA enhances sulfuric acid nucleation in the case of MA, it may suppress nucleation in the case of SA-TMA. Introducing MA into the SA-MSA system saw an increase in particle concentrations at higher concentrations of MSA, whereas TMA saw a decrease in particle concentrations at high MSA concentrations. However, in the atmosphere, MSA concentration typically never surpasses that of SA, so while the laboratory results show suppression in the case of TMA, the ratios of acids may not be atmospherically relevant.

Overall, results indicate that MSA is an important contributor to atmospheric nucleation and can increase particle formation rates for SA-base nucleation. Including MSA in models will be especially important as anthropogenic emissions of SO_2_ decrease, thus decreasing the [SA]/[MSA] ratio. These conclusions indicate that nucleation models that account for MSA-SA-base nucleation are required to better predict particle number concentrations in atmosphere, especially in the marine environment.

## Data Availability

The data underlying this study are openly available in the Index of Chamber Atmospheric Research in the United States (ICARUS) at (https://icarus.ucdavis.edu/experiment/869)

## Supporting Information Available

Results discussing vwCPC cut point experiments and vwCPC coagulation experiments (PDF)
