# Supplementary material for: Role of Methanesulfonic Acid in Sulfuric Acid–Amine and Ammonia New Particle Formation
Source: ACS Earth Space Chem. 2023 Mar 7;7(3):653–60. doi: 10.1021/acsearthspacechem.3c00017 (PMC10026172; doi:10.1021/acsearthspacechem.3c00017)
Supplement: Supplementary file 1 — sp3c00017_si_001.pdf [file sp3c00017_si_001.pdf]

Supporting Information For

Role of Methanesulfonic Acid in Sulfuric Acid-Amine and Ammonia New Particle Formation

Jack S. Johnson[1,2], Coty N. Jen[1,2]*

[1]*Department of Chemical Engineering, Carnegie Mellon University, Pittsburgh, PA, 15213, USA*

[2]*Center for Atmospheric Particle Studies, Carnegie Mellon University, Pittsburgh, PA, 15213, USA*

*Author to whom correspondence should be addressed (cotyj@andrew.cmu.edu)

Section 1: vwCPC cut point experiments

Figure S1 compares the particle concentrations measured by the vwCPC (TSI3789)[1] at a 1-nm and a 2-nm cut points when 7 pptv of trimethylamine (TMA) is injected into the flow reactor ([SA] = 6x10^{7} cm^{-3} and [MSA] = 2x10^{9} cm^{-3}). These results show that at a 2.4 s nucleation time, over 97% of the particles formed are 1 nm (assuming sharp cut points). Thus, a significant fraction of the particles formed during the nucleation experiments is freshly nucleated particles that have experienced little to no growth or coagulation.

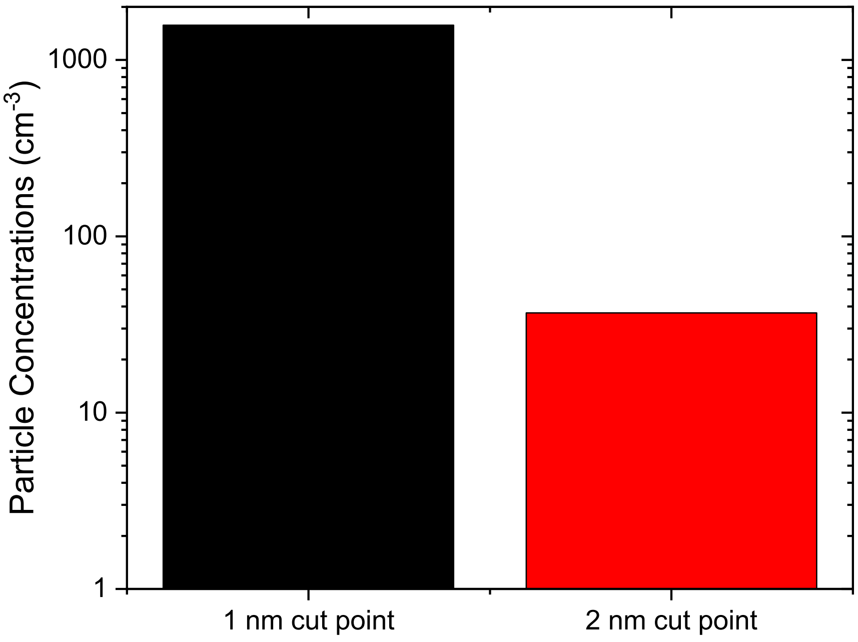

Figure S1: Comparison of particle concentrations at the 1-nm cut point vs. 2-nm cut point on the vwCPC. The black bar shows particle concentrations at the 1-nm cut point and the red bar at the 2-nm cut point. [TMA]=7 pptv and [SA]= 6x10^{7} cm^{-3} and [MSA] = 2x10^{9} cm^{-3}

Figure S2 provides evidence that SA-MSA-TMA particle concentration decrease with increasing [TMA] is not due to larger particles due to coagulation. This figure compares the particle concentrations from the vwCPC at a 1-nm cut point and a 2-nm cut point when 52 pptv of trimethylamine (TMA) is injected into the flow reactor ([SA] = 5x10^{7} cm^{-3} and [MSA] = 9x10^{8} cm^{-3}). These results show that particle counts drop over 90% in the SA-TMA system at the larger cut-point. In addition, 98% of the particles observed in the SA-MSA-TMA system are ~1 nm. Due to the significant drop in particle concentrations between cut-points, coagulation does not drive the decrease in particle concentrations when injecting MSA.

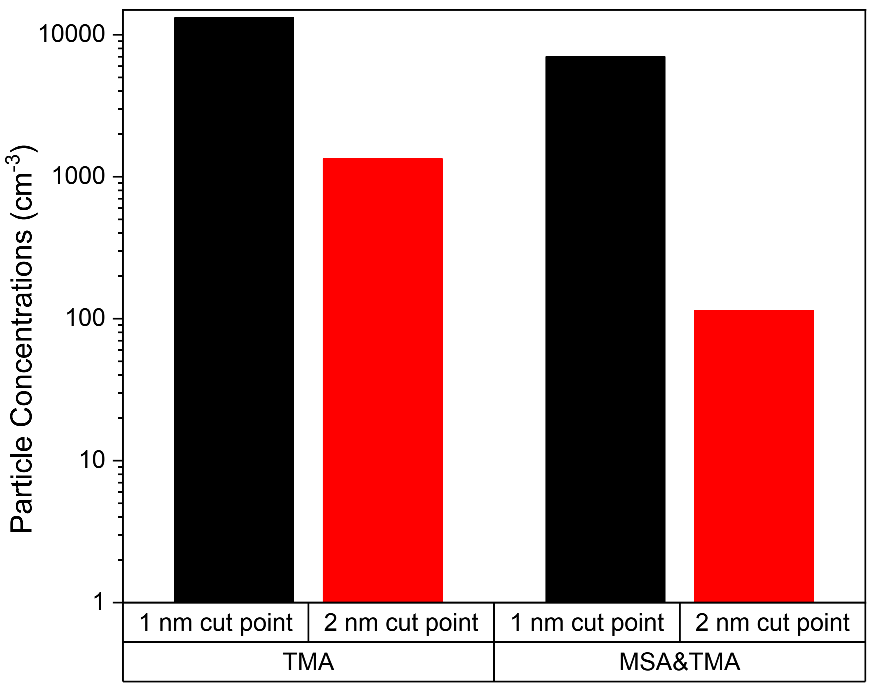

Figure S2 compares particle concentration at the 1-nm cut point vs. the 2-nm cut point on the vwCPC. The black bar shows particle concentrations at the 1 nm cut point and the red bar at the 2 nm cut point. The first set of bars shows SA+TMA, and the second set is SA-MSA-TMA. [TMA] is 52 pptv, [SA] is 5×10^{7} cm^{-3} and [MSA] is 9×10^{8} cm^{-3}.

Section 2: Computational Chemistry Results

Compiled binding free energies of uncharged clusters containing MSA, SA, and DMA from Elm and are given in Table S1.[2,3]

Table S1 Binding free energies from Elm of clusters pertinent to this study.[2,3]

**Clusters**	**Binding Free Energies (kcal/mol)**
SA·SA	-5.5
MSA·SA	-5.1
MSA·MSA	-5.4
SA·MA	-7.2
MSA·MA	-3.9
SA·DMA	-11.5
MSA·DMA	-7.1
SA·TMA	-12.6
MSA·TMA	-8.7
SA·SA·NH_3	-19.4
MSA·SA·NH_3	-18.3
SA·SA·NH_3·NH_3	-27.0
MSA·SA·NH_3·NH_3	-23.6
SA·SA·MA	-24.4
MSA·SA·MA	-24.2
SA·SA·MA·MA	-36.6
MSA·SA·MA·MA	-33.8
SA·SA·DMA	-29.4
MSA·SA·DMA	-28.2
MSA·SA·TMA	-24.9
SA·SA·TMA·TMA	-41.5
MSA·SA·TMA·TMA	-31.9

References:

(1) Hering, S. V.; Lewis, G. S.; Spielman, S. R.; Eiguren-Fernandez, A.; Kreisberg, N. M.; Kuang, C.; Attoui, M. Detection near 1-Nm with a Laminar-Flow, Water-Based Condensation Particle Counter. *Aerosol Science and Technology* **2017**, *51* (3), 354–362. https://doi.org/10.1080/02786826.2016.1262531.

(2) Elm, J. Clusteromics III: Acid Synergy in Sulfuric Acid–Methanesulfonic Acid–Base Cluster Formation. *ACS Omega* **2022**, *7* (17), 15206–15214. https://doi.org/10.1021/acsomega.2c01396.

(3) Elm, J. Clusteromics II: Methanesulfonic Acid–Base Cluster Formation. *ACS Omega* **2021**, *6* (26), 17035–17044. https://doi.org/10.1021/acsomega.1c02115.
